# Structure-function analysis of plant G-protein regulatory mechanisms identifies key Gα-RGS protein interactions

**DOI:** 10.1016/j.jbc.2024.107252

**Published:** 2024-04-01

**Authors:** Maria Daniela Torres-Rodriguez, Soon Goo Lee, Swarup Roy Choudhury, Rabindranath Paul, Balaji Selvam, Diwakar Shukla, Joseph M. Jez, Sona Pandey

**Affiliations:** 1 Donald Danforth Plant Science Center, St Louis, Missouri, USA; 2 Department of Molecular & Cellular Biology, Kennesaw State University, Kennesaw, Georgia, USA; 3 Department of Biology, Indian Institute of Science Education and Research, Tirupati, India; 4 Department of Chemical and Biomolecular Engineering, University of Illinois at Urbana-Champaign, Urbana, Illinois, USA; 5 Department of Biology, Washington University in St Louis, St Louis, Missouri, USA

**Keywords:** Gα protein, RGS, plant, rice, activation/deactivation, structure, Selaginella moellendorffi

## Abstract

Heterotrimeric GTP-binding protein alpha subunit (Gα) and its cognate regulator of G-protein signaling (RGS) protein transduce signals in eukaryotes spanning protists, amoeba, animals, fungi, and plants. The core catalytic mechanisms of the GTPase activity of Gα and the interaction interface with RGS for the acceleration of GTP hydrolysis seem to be conserved across these groups; however, the *RGS* gene is under low selective pressure in plants, resulting in its frequent loss. Our current understanding of the structural basis of Gα:RGS regulation in plants has been shaped by Arabidopsis Gα, (AtGPA1), which has a cognate RGS protein. To gain a comprehensive understanding of this regulation beyond Arabidopsis, we obtained the x-ray crystal structures of *Oryza sativa* Gα, which has no RGS, and *Selaginella moellendorffi* (a lycophyte) Gα that has low sequence similarity with AtGPA1 but has an RGS. We show that the three-dimensional structure, protein-protein interaction with RGS, and the dynamic features of these Gα are similar to AtGPA1 and metazoan Gα. Molecular dynamic simulation of the Gα-RGS interaction identifies the contacts established by specific residues of the switch regions of GTP-bound Gα, crucial for this interaction, but finds no significant difference due to specific amino acid substitutions. Together, our data provide valuable insights into the regulatory mechanisms of plant G-proteins but do not support the hypothesis of adaptive co-evolution of Gα:RGS proteins in plants.

Heterotrimeric G-proteins are universal signaling conduits in all eukaryotic groups, ranging from yeast and humans to algae and vascular plants (1, 2). They regulate a variety of cellular processes involving millisecond-level responses, such as perception of light or neurotransmission in humans, to slower lifetime-level developmental aspects, such as body plan and architecture in plants (3, 4, 5). In all organisms, the core G-protein complex consists of one subunit each of the α, β, and γ proteins and exists in bimodal active *versus* inactive states. The transition between the active and inactive states is driven by the nucleotide-bound form of the Gα subunit (6, 7). In the resting state, Gα is GDP-bound and associated with the Gβγ dimer. Signaling is initiated when the bound GDP is exchanged for GTP on Gα, which results in conformational changes that dissociate the heterotrimeric complex into Gα·GTP and Gβγ. Both entities can interact with downstream effectors to transduce the signal. The inherent GTPase activity of Gα hydrolyzes the bound GTP, regenerating its GDP-bound form, which due to its high affinity for Gβγ, reconstitutes the inactive heterotrimer (8, 9).

Although the basic structure and key biochemical activities of the individual proteins of the G-protein complex are conserved across phyla, their regulation during activation and deactivation, both in terms of the identity of regulatory proteins and the underlying mechanisms exhibit notable differences. In metazoan systems, activation typically occurs due to the guanine nucleotide exchange factor (GEF) activity of a cognate G-protein coupled receptor (GPCR) (10). The absence of a GEF activity possessing GPCR in plants has led to the hypothesis that the plant Gα is self-activated (11). This is suggested by *in vitro* biochemical quantification of the nucleotide exchange rate of *Arabidopsis thaliana* GPA1 (AtGPA1), which is ∼50-fold faster than the fastest human Gα protein (12). The fast nucleotide exchange activity of AtGPA1 has been proposed to be due to the difference in its conformational dynamic properties compared to metazoan Gα (13, 14). Furthermore, alternative activation mechanisms, such as phosphorylation-based de/activation by plant-specific receptor-like kinases, have also been suggested for plant Gα proteins; however, their details remain unexplored (15, 16, 17, 18).

The Gα are slow GTPases—their rate of GTP hydrolysis is significantly lower than the rate of GTP binding. To avoid response saturation by active Gα proteins, faster deactivation is achieved with the help of GTPase activity accelerating proteins (GAP), such as the regulator of G-protein signaling (RGS) (19), which binds to activated Gα to accelerate GTP-hydrolysis. The catalytic mechanism of RGS-mediated Gα deactivation and their interaction dynamics and specificities have been characterized at the atomic level in metazoan systems. However, in plants, the existing data on RGS-mediated deactivation of Gα presents a complex picture. Compared to their metazoan counterparts, plant Gα has faster GDP/GTP exchange and remarkably slower GTP hydrolysis - at least an order of magnitude slower than the slowest mammalian Gα homologs (12, 20), suggesting that they will remain GTP-bound unless deactivated with the help of the GAP activity of RGS proteins (12, 13, 20). This implies that the RGS-dependent deactivation step is central to the regulation of the plant G-protein signaling cycle (12, 20). Biochemical studies in Arabidopsis support this hypothesis, to some extent. Intriguingly, the RGS coding genes are under low selective pressure in plants and many genomes do not possess any RGS (2, 21), even though the G-protein signaling is functional in these plants.

The structural basis of how Gα interacts with RGS to accelerate GTPase activity is established in metazoan systems (22, 23, 24, 25). The two domains of the Gα—the Ras-like domain and the helical domain (HD)—flank the guanine nucleotide binding site (8). The Ras-like domain hydrolyses GTP and provides the binding surfaces for the Gβγ dimer, RGS, and other effector proteins (6). It contains three conformationally dynamic regions, named switches I, II, and III, which undergo structural changes in the GDP- *versus* GTPγS-bound forms of Gα (8, 9). RGS binds to the switch regions on the GTP-bound Gα causing conformational changes around the active site that stabilize Gα for GTP hydrolysis (26). Residues located in the HD of metazoan Gα also participate in the interaction with RGS and provide specificity for its cognate partners (25, 27).

To date, there is only one available crystal structure of a plant Gα protein, the *Arabidopsis* Gα (AtGPA1), in its GTP-bound conformation (13). The structure of a plant RGS protein has not been elucidated, and the little information that exists is based on molecular dynamics (MD) simulations of the predicted *Arabidopsis* and *Setaria italica* GPA1:RGS1 complexes (21, 28). These models have been used to explain the loss of RGS coding genes in specific plant groups. It suggests that the replacement of a hydroxyl-containing threonine in the switch I region of the Ras-like domain with asparagine in a subset of plant Gα destabilizes the interaction interface, leading to loss of interaction and ultimately loss of RGS itself (11). This model also proposes an adaptive coevolution of Gα:RGS pairs in specific plants (*e.g.*, *S. italica*), where the destabilizing threonine to asparagine replacement in Gα was compensated for by a corresponding change in its cognate RGS protein, allowing for Gα:RGS interaction (28). Comparative sequence analysis of Gα and RGS proteins from across the plant phylogeny did not support the specific amino acid replacement-based model of Gα:RGS interaction (21). Furthermore, *in vitro* experiments showed that an RGS protein from any species (including humans) can accelerate the GTPase activity of any plant Gα, including those without a native RGS, and the interaction interface of Gα:RGS is conserved across phyla (21). This was confirmed using *in vivo* experiments that introduced the *RGS* gene into plants without native RGS. Comparative characterization of transgenic *Brachypodium* (no *RGS* coding gene in genome) and *Setaria* (with *RGS* coding gene in genome) plants demonstrated that the regulation of G-protein-dependent phenotypes (*e.g.*, control of internode elongation) in both these plants is similar (29). Moreover, native or gain-of-function RGS mutants exhibited similar effects on *in planta* regulation of the G-protein cycle, *i.e.*, overexpression of *RGS* in Brachypodium resulted in plant phenotypes similar to what is observed due to the loss of Gα function (29).

Not only is the distribution of Gα:RGS proteins variable within plant groups (*e.g.*, all eudicots have both Gα and RGS, many monocots do not have RGS, and many bryophytes lack both Gα and RGS (2)), but there are considerable variations in their biochemistries and functions. Moreover, it is not known whether the three-dimensional structure of a Gα from a plant that has no RGS protein differs from those that have an RGS protein. Similarly, the non-vascular plant Gα shows relatively limited sequence similarity with AtGPA1 and can only complement a subset of phenotypes of Arabidopsis Gα (*gpa1*) mutants, which suggests their functional diversification (30). The extent to which these differ at the structural level is not known. To gain a broader perspective on the regulation of Gα in plants, we performed a structure-function analysis of Gα proteins from a lycophyte *Selaginalla moellendorffi* (SmGPA), a member of the earliest diverging group of modern vascular plants, and a monocot *Oryza sativa* (rice, OsRGA1), which does not have an *RGS* gene in its genome. Our results show conserved and divergent structural characteristics of Gα across a broad range of plant species and demonstrate how these might affect its regulation by RGS.

## Results

### Three-dimensional structures of Selaginella (a lycophyte) and rice (a monocot) Gα

**Figure 1:**
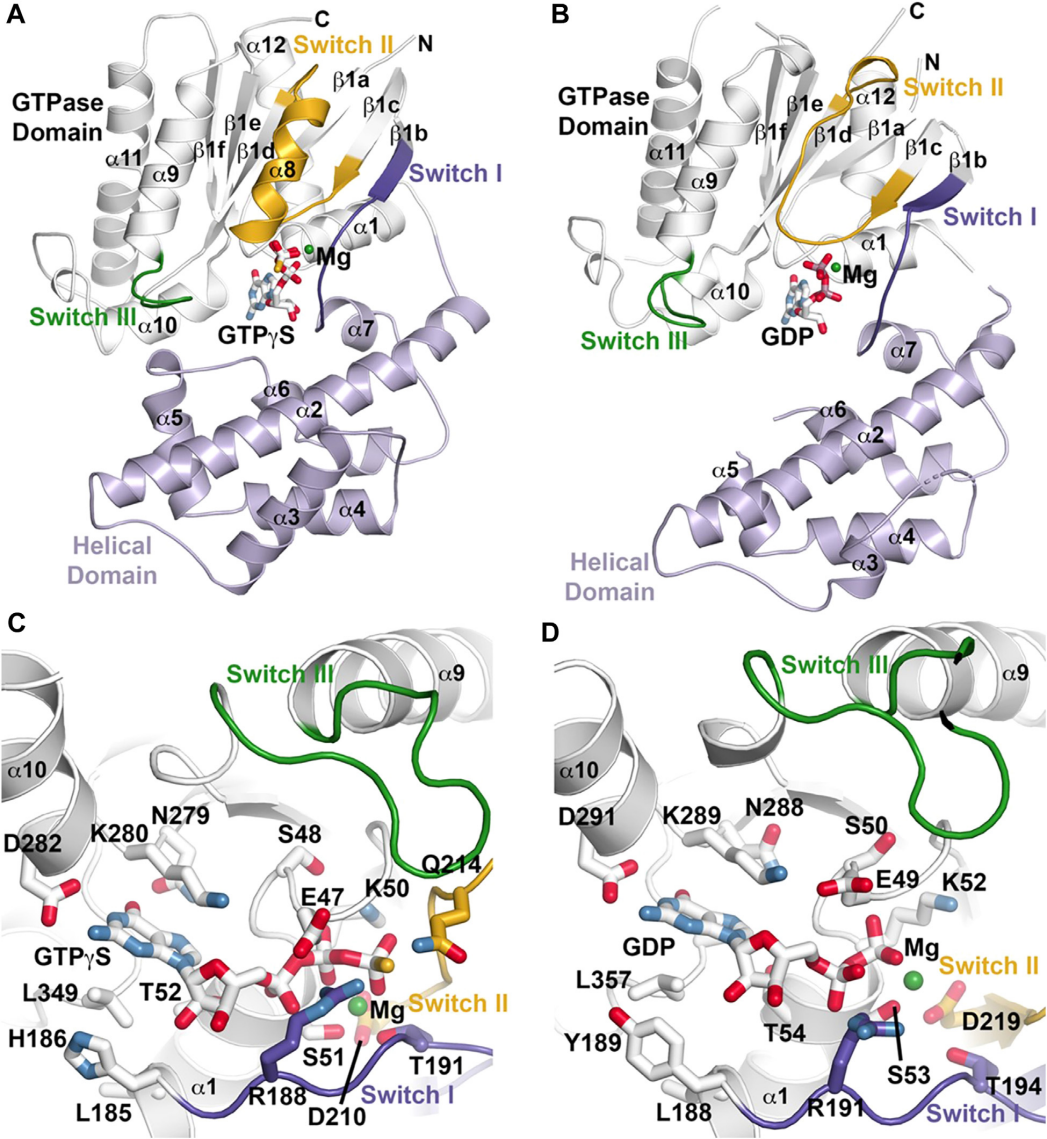
**Three-dimensional structures of SmGPA•GTPγS and OsRGA1·GDP complexes.***A*, ribbon diagram of SmGPA complexed with GTPγS and Mg^2+^. The Ras-like GTPase domain and helical domains are colored *white* and light *purple*, respectively. The three switch regions are colored dark *purple*, *gold*, and *green*, respectively. *B*, ribbon diagram of OsRGA1 complexed with GDP and Mg^2+^. Coloring of the two domains and three switch regions are as in *panel A*. *C*, SmGPA•GTPγS active site view. Active site residues contacting GTPγS and Mg^2+^ (*green sphere*) are shown as stick models. Loops and residues of switch I (*dark purple*), switch II (*gold*), and switch III (*green*) and shown. *D*, OsRGA1·GDP active site view. Active site residues contacting ADP and Mg^2+^ (*green sphere*) are shown as stick models with the three switch regions shown and colored as in *panel C*.

The x-ray crystal structures of the SmGPA·GTPγS and OsRGA1·GDP complexes were solved at 2.57 Å and 2.99 Å resolution, respectively, by molecular replacement using AtGPA1 as the search model (Table 1). SmGPA and OsRGA1 share 55.9% and 77.5% amino acid sequence identity, respectively, with AtGPA1 and 50.5% identity with each other. SmGPA crystallized with two monomers in the asymmetric unit and OsRGA1 crystallized with four monomers in the asymmetric unit. SmGPA and OsRGA1 share similar bi-domain structures observed in other eukaryote Gα proteins (8, 9, 10, 13). A three-dimensional structure similarity search using the DALI server identifies multiple Gα proteins, including AtGPA1 (13), human and murine Gα13 (31, 32), human Gs (33), *Entamoeba histolytica* Gα (34), human Gq (35), and human Gi (36), that share 30 to 56% amino acid identity with both SmGPA and OsRGA1, as well as the conservation of three-dimensional fold with 1.6 to 2.8 Å r.m.sd for 225 to 372 C_α_-atoms in these enzymes from varied organisms. The Ras-like GTPase domain of SmGPA with the GTP analog consists of five α-helices (α1, α9-12) surrounding a six-stranded β-sheet (β1a-b1f) with the helical domain (α2-α7) connected by two linking segments (Fig. 1*A*). The rice protein is similar; however, there are structural changes that correspond to the GDP-bound form (Fig. 1*B*), as discussed below. An unambiguous electron density for the guanine nucleotide ligand was observed in each structure (Fig. S1, *A* and *B*). These provide structural information on the active (GTP-bound) and inactive (GDP-bound) forms of plant Gα-proteins. The superimposition of the three-dimensional structures of SmGPA, OsRGA1, AtGPA1, and other plant Gα proteins (predicted using Alphafold) representing the plant evolutionary scale, show a high degree of overlap of the overall structure, particularly in the Ras-like GTPase domain comprising the switch regions (Fig. S2), suggesting that the ancestral Gα core is conserved throughout the plant evolutionary lineage.

**Table 1:** Summary of crystallographic statistics for SmGPA and OsRGA1 structures

Crystal	SmGPA·GTPγS·Mg^2+^	OsRGA1·GDP·Mg^2+^
Space group	P2_1_	P2_1_
Cell dimensions	*a* = 109.1 Å, *b* = 61.47 Å, c = 111.9 Å; b = 115.2°	*a* = 68.39 Å, *b* = 68.42 Å, c = 168.0 Å; b = 89.9°
Data Collection		
Wavelength	0.979 Å	0.979 Å
Resolution range (highest shell)	42.6–2.57 Å (2.61–2.57 Å)	48.4–2.99 Å (3.04–2.99 Å)
Reflections (total/unique)	157,952/42,531	119,365/31,650
Completeness (highest shell)	99.8% (100%)	99.9% (100%)
<I/σ> (highest shell)	16.6 (2.0)	26.4 (1.9)
R_sym_ (highest shell)	8.4% (78.2%)	5.2% (61.0%)
Refinement		
R_cryst_/R_free_	16.6%/21.1%	26.6%/28.4%
No. of protein atoms	5496	9801
No. of waters	141	-
No. of ligand atoms	66	116
R.m.s. deviation, bond lengths	0.008 Å	0.007 Å
R.m.s. deviation, bond angles	1.092°	1.639°
Avg. B-factor: protein, water, ligand	57.9, 42.7, 51.3 Å^2^	97.4–82.7 Å^2^
Stereochemistry: favored, allowed, outliers	97.0, 2.8, 0.2%	92.8, 4.2, 3.0%

### Comparison of active (GTPγS-bound) and inactive (GDP-bound) forms of plant Gα

In the SmGPA·GTPγS complex structure (Figs. 1, *A* and *C*, and S1*C*), the guanine nucleotide analog is bound in a highly conserved active site to provide a view of the active GTPase located between the Ras-like GTPase and the helical domains (8). The guanine ring of GTPγS is bound through interactions with Asn279, Lys280, Asp282, Ala384, and Leu349, and the ribose hydroxyl groups hydrogen bond with the backbone carbonyl of His186. Residues of the canonical diphosphate binding loop (*i.e.*, P-loop - Glu47, Ser48, Gly49, Ser51, Thr52), as well as the Mg^2+^ ion and its interacting side-chains (Ser51, Thr191, Asp210), position the γ-phosphate for hydrolysis. In the active GTPase configuration of SmGPA, the three dynamic "switch" regions (switch I - Ala187-Val198; switch II - Val208-Ala230; switch III - Asp241-Arg252) involved in GTP binding and hydrolysis encompass the terminal group of bound GTPγS. In addition to the amine group of Lys50, Asp210 and Gln214 in switch II orient the terminal phosphate. Asp210 does this through interaction with the Mg^2+^ ion and Gln214 through hydrogen bonding with the γ-phosphate. Hydrogen bonding between the side-chain hydroxyl group of Thr191 and the γ-phosphate locks the switch I loop to bring Arg188 into position for the hydrolysis reaction. Beyond the active site, residues in switch III interact with residues in the helical domain to bring the two halves of SmGPA together.

In comparison, the OsRGA1·GDP complex structure (Figs. 1, *B* and *D*, and S1*D*) highlights how changes in the active site following GTP hydrolysis result in large-scale conformational change. As observed in other G-protein structures (8, 9, 10), major structural differences are observed in the three switch regions. Within the active site of the OsRGA1·GDP complex, interactions with the guanine ring and ribose of GDP are comparable to GTPγS bound in SmGPA, even with substitution of Tyr189 in OsRGA1 for the corresponding His186 of SmGPA. Although the backbone of the P-loop maintains position, multiple side chains from P-loop residues, such as Glu49, Ser50, and Lys52 in OsRGA1, shift. More pronounced changes occur in the three switch regions around the empty γ-phosphate site.

For switch I, the loss of the terminal phosphate breaks the key binding and catalytic interactions. In the OsRGA1·GDP structure, Thr194 (Thr191 in SmGPA) lacks an interaction partner and the catalytically critical Arg191 guanidino side-chain (Arg188 in SmGPA) points away from the active site. The loss of interactions with the bound ligand shifts the position of the β1b-α7 loop in switch I, which includes Arg191 and Thr194, away from the GTPase domain. This contributes to an altered orientation of the helical domain. The largest tertiary structural changes occur in switch II, as the well-organized α8 helix of this region in the SmGPA·GTPγS complex (Fig. 1*A*) undergoes major conformational changes in the OsRGA1·GDP complex (Fig. 1*B*). Of the four OsRGA1 molecules in the asymmetric unit, three lacked electron density for residues corresponding to α8. Finally, the interaction between residues in switch III and the helical domain is broken, as the latter shifts away from the GTPase domain. This opens the active site for the release of GDP in the G-protein cycle (8, 9, 10).

**Figure 2:**
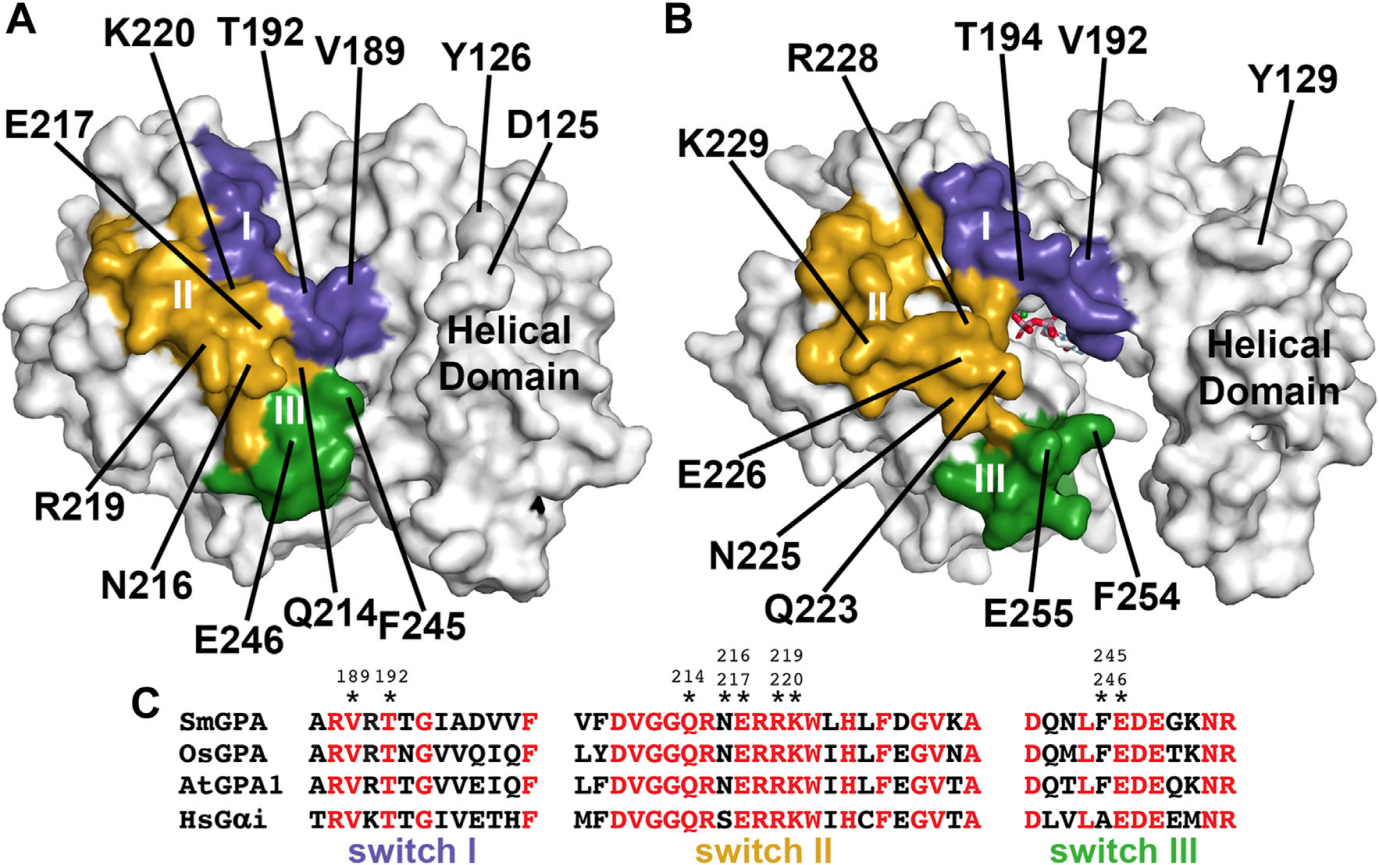
**RGS interaction regions in GTP and GDP-bound forms of Gα.** Surface rendering of the SmGPA·GTPγS (*A*) and OsRGA1·GDP (*B*) complexes showing the RGS interaction region. Residues of switch I (*dark purple*), switch II (*gold*), and switch III (*green*) are colored with selected corresponding residues indicated. *C*, targeted sequence alignment of the three switch regions of SmGPA, OsRGA1, AtGPA1, and human (Hs) Gαi. Positions in SmGPA targeted for site-directed mutagenesis are indicated by an asterisk with a number above.

Conformational changes between the GTP- and the GDP-bound forms of Gα also impact interaction with RGS proteins (22, 26). Comparison of the SmGPA·GTPγS and OsRGA1·GDP complex structures (Fig. 2, *A* and *B*) highlights changes in the switch regions, as well as the helical domain, that form the interaction surface with RGS. Previous work demonstrated that the Gα-RGS interface is conserved across species from plants to mammals (21), even in Gα proteins from plants lacking RGS proteins. For example, the amino acid sequences of each switch region in SmGPA, OsRGA1, AtGPA1, and human Gαi contain multiple invariant residues (Fig. 2*C*). This conservation allows for the functional compatibility of Gα and RGS proteins across kingdoms and implies that the conformation of the GTP-bound form provides a platform for interaction with the RGS protein (21, 22, 26).

### Role of the switch regions in Selaginella Gα-RGS protein interaction

Although the structure of a plant Gα-RGS complex remains to be determined, previous modeling of the Arabidopsis GPA1·RGS complex (21), based on the x-ray crystal structure of the human G_iα1_·RGS4 complex (22), identifies amino acids in SmGPA that may contribute to SmRGS interaction (Fig. 2*A*). These residues include Val189, Thr192, and Lys220 in switch I; Gln214, Asn216, Glu217, and Arg219 in switch II; Phe245 and Glu246 in switch III; and Asp125 and Tyr126 in the helical domain (Fig. 2, *A* and *C*). To probe their potential contribution to SmGPA·SmRGS interaction, a series of alanine scanning point mutants were generated (D125A, Y126A, V189A, T192A, Q214A, N216A, E217A, R219A, K220A, F245A, and E246A) and used for yeast two-hybrid, GTPase, and ITC analyses. Each mutant was expressed and purified to yields and purity comparable to wild-type SmGPA.

**Figure 3:**
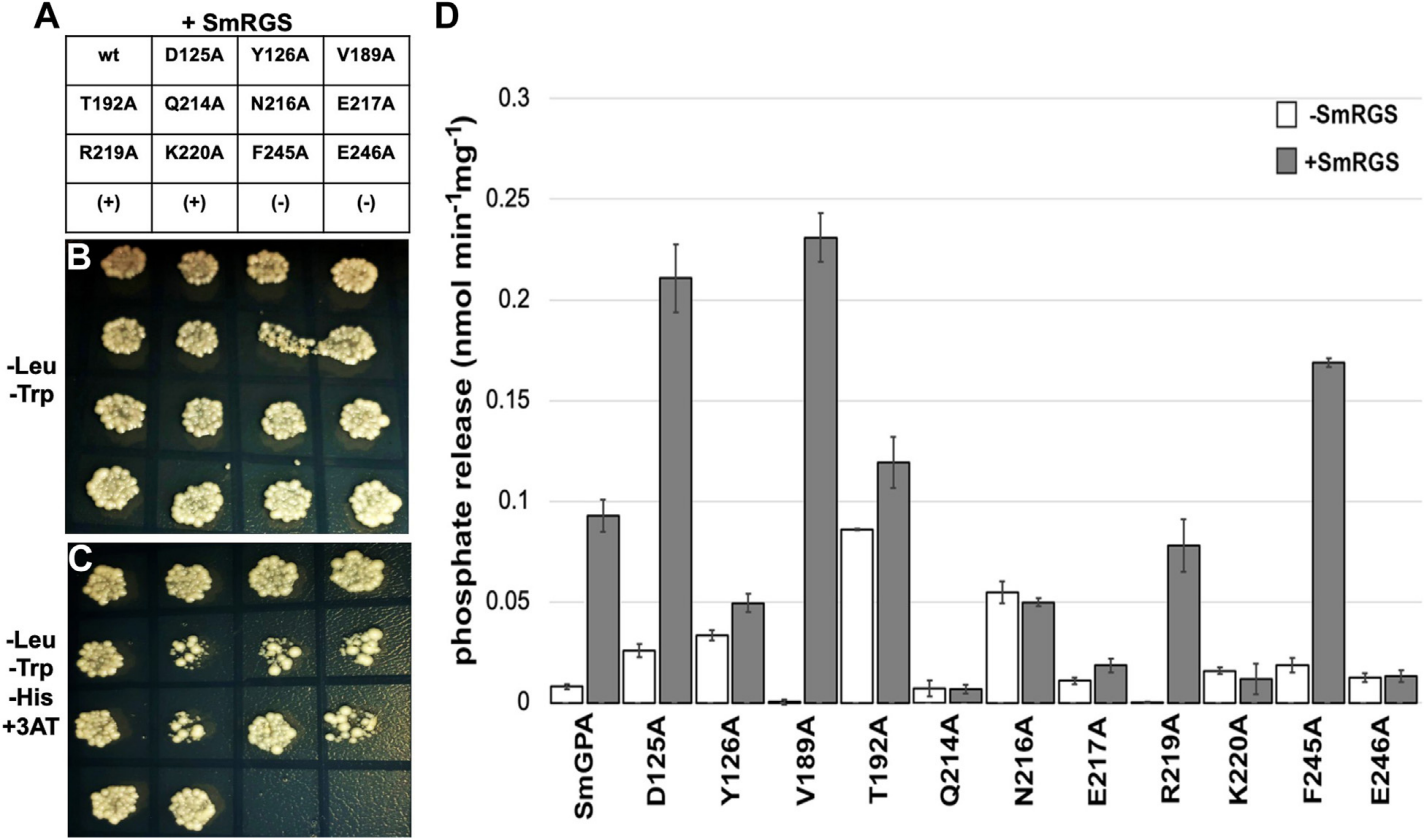
**SmGPA interaction analysis.***A*, the interaction between wild-type and mutant SmGPA (noted in panel) and SmRGS were examined using a yeast two-hybrid assay with co-transformed *S. cerevisiae* growing on either control (*B*) or selective medium (*C*). *D*, GTPase activity of wild-type and mutant SmGPA measured by phosphate release in the absence (white bars) or presence (*gr*e*y bars*) of SmRGS. Data are shown as the mean ± SE (n = 3).

In the yeast two-hybrid assay (Fig. 3, *A*–*C*), five SmGPA mutants (Q214A, N216A, E217A, K220A, and E246A) exhibit reduced growth under selective conditions, which suggests disruption of binding with SmRGS *in vivo*. Similarly, a phosphate release assay that measures GTPase activity indicates that the same five SmGPA mutants did not increase GTPase activity in the presence of SmRGS (Fig. 3*D*). On the contrary, wild-type SmGPA, as well as the D125A, Y126A, V189A, T192A, R219A, and F245A SmGPA mutants, showed interaction and are activated by SmRGS, although some differences in activation levels were also observed compared to wild-type SmGPA (Fig. 3*D*).

**Figure 4:**
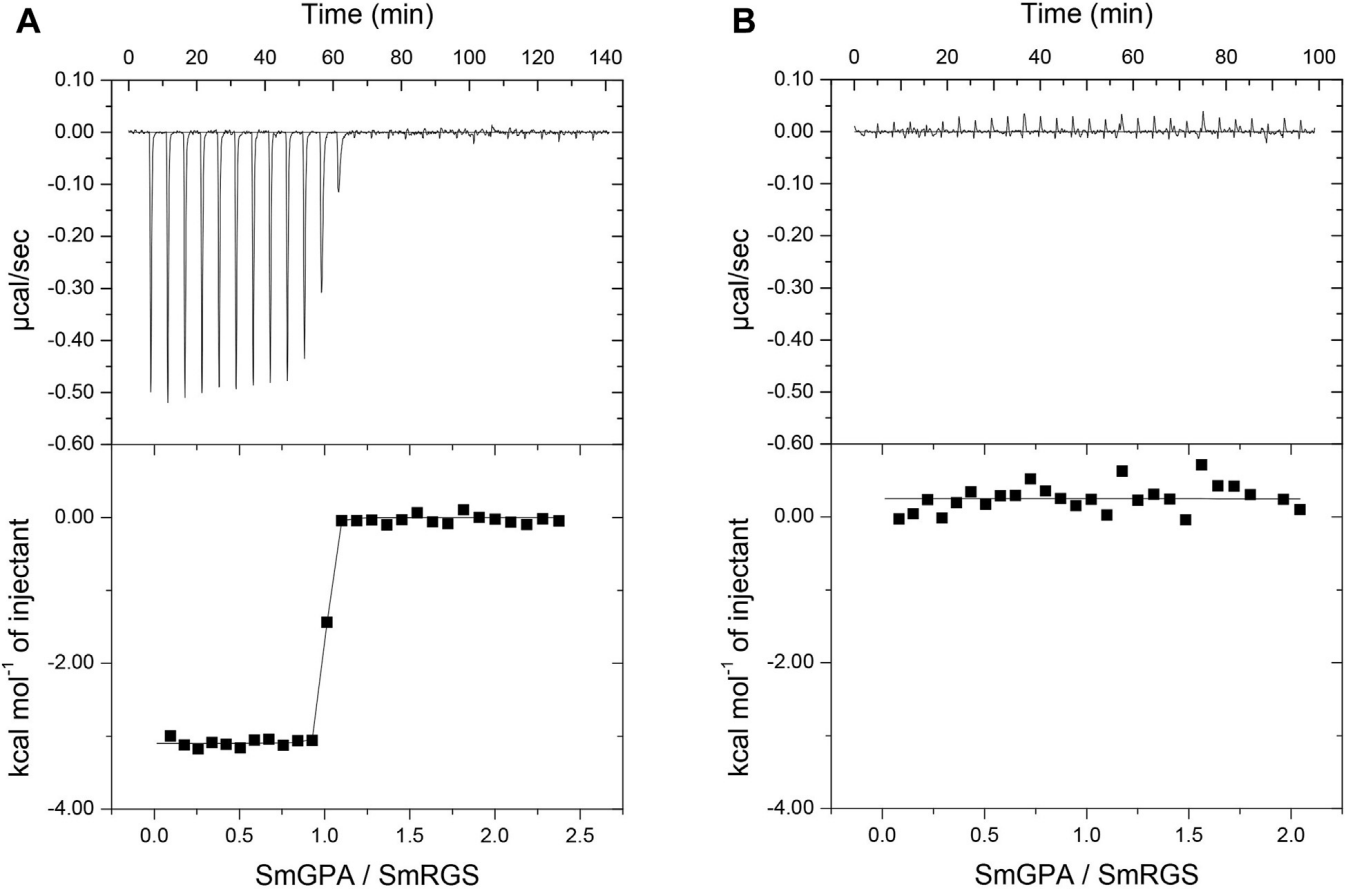
**Isothermal titration calorimetry (ITC) analysis of SmGPA·SmRGS interaction.** Titrations of SmRGS with SmGPA prepared with GDP, Mg^2+^, AlCl_3_, and 20 mM NaF to mimic the activated form (*A*) and with GDP alone (*B*) are shown. In each panel, the upper window shows the ITC data plotted as head signal (mcal/s) *versus* time (min) with the lower panel showing the integrated heat response per injection normalized as heat per mole of injectant.

To quantitatively assess the interaction of SmGPA and SmRGS, ITC was used. Wild-type and mutant SmGPA were prepared using GDP, Mg^2+^, AlCl_3_, and NaF, which leads to formation of a GDP·Mg^2+^·AlF_4_^−^ complex that mimics the GTP-bound form (22). ITC analysis of SmGPA·SmRGS binding (Fig. 4*A*; Table 2) indicates a tight (*K*_d_ = 23 nM) binding interaction. Titration using SmGPA prepared with GDP and Mg^2+^ shows no interaction with SmRGS (Fig. 4*B*), as there was no observable heat signature. Analysis of the interaction of the D125A, Y126A, V189A, T192A, Q214A, N216A, E217A, R219A, K220A, F245A, and E246A SmGPA mutants with SmRGS was performed (Table 2). Three SmGPA mutants (T192A, E217A, and K220A) showed no detectable interaction. The Q214A (12-fold), R219A (85-fold), F245A (735-fold), and E246A (800-fold) SmGPA mutants displayed 12- to 800-fold decreases in binding affinity. Alanine substitutions of Asp125, Tyr126, Val189, and Asn216 had minor (<5-fold) changes in the *K*_d_ of SmGPA·SmRGS interaction.

**Table 2:** Thermodynamic parameters of SmRGS binding to wild-type and mutant SmGPA

SmGPA	*K*_d_ (nM)	ΔG (kcal mol^−1^)	ΔH (kcal mol^−1^)	-TΔS (kcal mol^−1^)
wild-type	23 ± 3	−11.7 ± 0.4	−3.07 ± 0.03	−8.65 ± 0.44
D125A	86 ± 22	−10.8 ± 0.1	−6.53 ± 0.11	−4.31 ± 0.28
Y126A	59 ± 3	−11.0 ± 0.1	−7.45 ± 0.04	−3.59 ± 0.07
V189A	130 ± 10	−9.57 ± 1.00	−2.89 ± 0.39	−6.69 ± 1.38
T192A	--	--	--	--
Q214A	270 ± 10	−10.2 ± 1.2	−5.30 ± 0.59	−4.93 ± 1.88
N216A	55 ± 2	−11.2 ± 0.4	−4.81 ± 0.17	−6.43 ± 0.63
E217A	--	--	--	--
R219A	1960 ± 240	−8.99 ± 0.11	−6.29 ± 0.27	−2.65 ± 0.28
K220A	--	--	--	--
F245A	16,900 ± 5400	−7.88 ± 0.11	3.10 ± 0.28	−10.9 ± 0.1
E246A	18,400 ± 220	−7.68 ± 0.51	−2.73 ± 0.81	−4.95 ± 0.87

Titrations were performed at 20 °C with SmGPA proteins prepared with GDP and NaF, as described in the Experimental Procedures. ITC data were fit to a one-site binding model with parameters shown as mean ± SE (n = 3).

### Targeted mutations of SmRGS disrupt interaction with SmGPA

**Figure 5:**
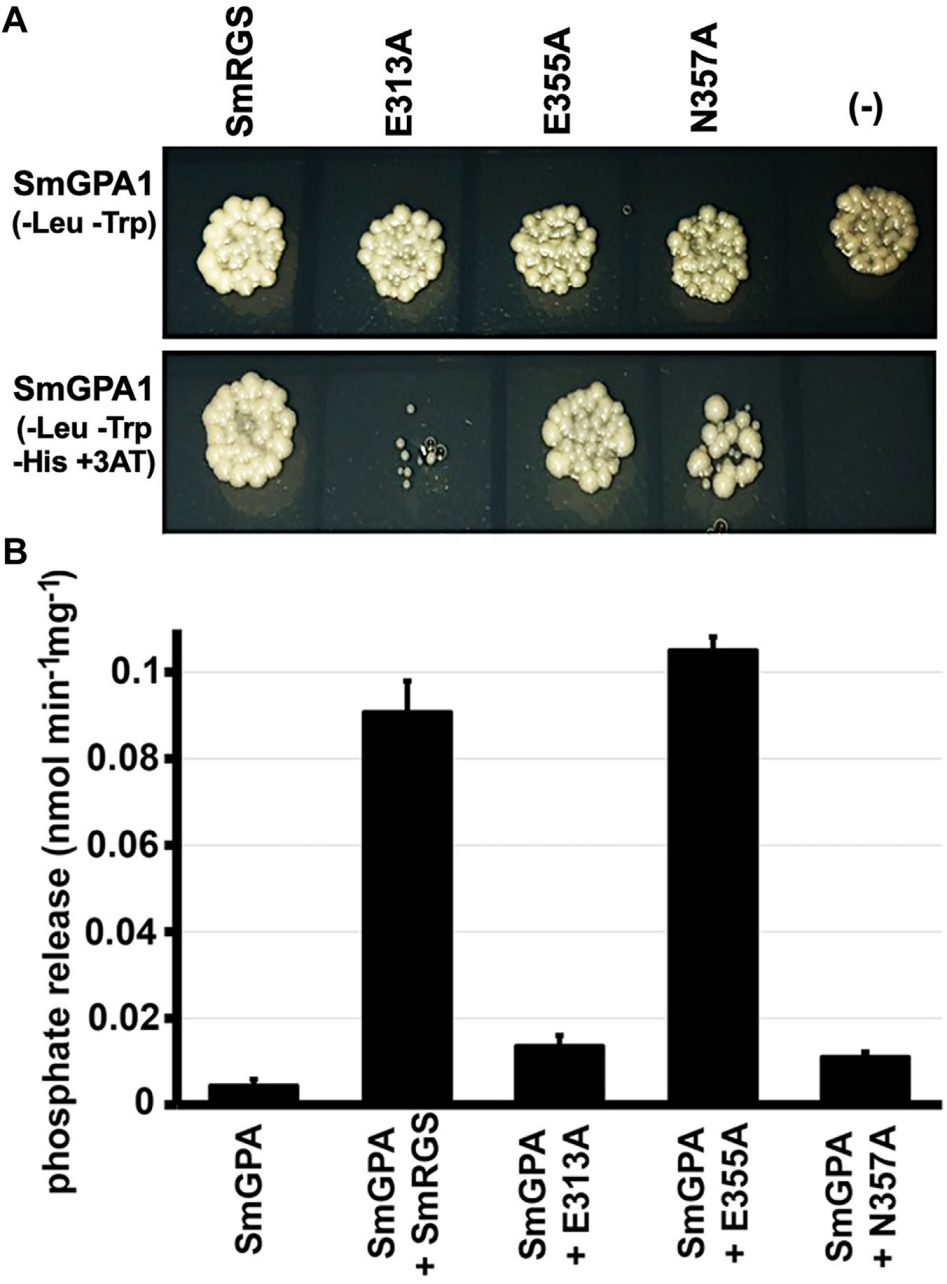
**SmRGS interaction analysis.***A*, interaction between wild-type and mutant SmRGS (noted in panel) and SmGPA was examined using a yeast two-hybrid assay with co-transformed *S. cerevisiae* growing on either control (*top*) or selective medium (*bottom*). *B*, GTPase activity of wild-type and mutant SmGPA measured by phosphate release in the absence or presence of wild-type or mutant SmRGS. Data are shown as the mean ± SE (n = 3).

In the x-ray crystal structure of the human Gα-RGS protein complex (22), a patch of acidic residues (corresponding to Glu313, Glu355, and Asn357 in SmRGS) on the RGS protein contact a lysine and glutamate (corresponding to Glu217 and Lys220 in SmGPA) on the Gα. Given that mutations at these two positions disrupt the SmGPA·SmRGS interaction and activation, three site-directed mutants of SmRGS (E313A, E355A, and N357N) were generated and assayed using for interaction using yeast two-hybrid, phosphate release assay, and ITC. Each of the three SmRGS mutants was expressed and purified to yields and purity comparable to those of wild type. The SmRGS E313A and N357A mutants exhibited impaired growth in the yeast two-hybrid assay (Fig. 5*A*). Similarly, these two SmRGS mutants did not increase the GTPase activity of SmGPA to levels observed with wild-type and E355A SmGPA (Fig. 5*B*). ITC analysis yielded comparable binding affinities for wild-type and E355A SmRGS (Table 3); however, the E313A and N357A SmRGS mutants decreased the binding affinity for SmGPA by 45- and 295-fold, respectively.

**Table 3:** Thermodynamic parameters of SmGPA binding to wild-type and mutant SmRGS

SmRGS	*K*_d_ (nM)	ΔG (kcal mol^−1^)	ΔH (kcal mol^−1^)	-TΔS (kcal mol^−1^)
wild-type	23 ± 3	−11.7 ± 0.4	−3.07 ± 0.03	−8.65 ± 0.44
E313A	1050 ± 60	−8.01 ± 0.04	−3.74 ± 0.06	−4.27 ± 0.12
E355A	16 ± 3	−10.5 ± 0.1	−7.04 ± 0.10	−3.42 ± 0.20
N357A	6770 ± 2750	−6.99 ± 0.24	−4.36 ± 0.63	−11.3 ± 0.4

Titrations were performed at 20 °C with SmRGS into SmGPA prepared with GDP and NaF, as described in the Experimental Procedures. ITC data were fit to a one-site binding model with parameters shown as mean ± SE (n = 3).

### Dynamics of plant Gα-RGS protein complexes

To explore the structural basis of the absence of RGS proteins in certain plant lineages, a previous report proposed a correlation between the replacement of a threonine (Thr194 in AtGPA1; Thr192 in SmGPA) with an asparagine (*e.g.*, Asn 195 in OsRGA1) as the causal substitution that led to the loss of *RGS* in several grass species (11). Substitutions are common at this site in plant Gα, with asparagine being a prevalent substitution, but there is no clear correlation between this substitution and the presence/absence of RGS (21). Although Thr192 substitution did not show an effect on interaction with SmRGS in yeast-2-hybrid assays, no interaction between these proteins was detected using ITC assays (Fig. 3, *A*–*C*, Table 2). In GTPase activity assays, the Thr192 substituted Gα showed an inherently higher GTPase activity, but no effect of RGS (Fig. 3*D*). Given the importance of this residue and the contradictory results with Y2H and ITC assays, we decided to further investigate its role in defining the Gα:RGS complex formation using MD simulations. Additionally, it has been proposed that the effect of Thr to Asn replacement in Gα can be compensated by Thr321 to Ser substitution in the corresponding RGS protein, with foxtail millet (*S. italica*) as an example, which is a grass species with an *RGS* gene in its genome (28). Therefore, we compared the MD simulations of the *S. italica* SiGPA:SiRGS complex with the AtGPA1:AtRGS complex.

**Figure 6:**
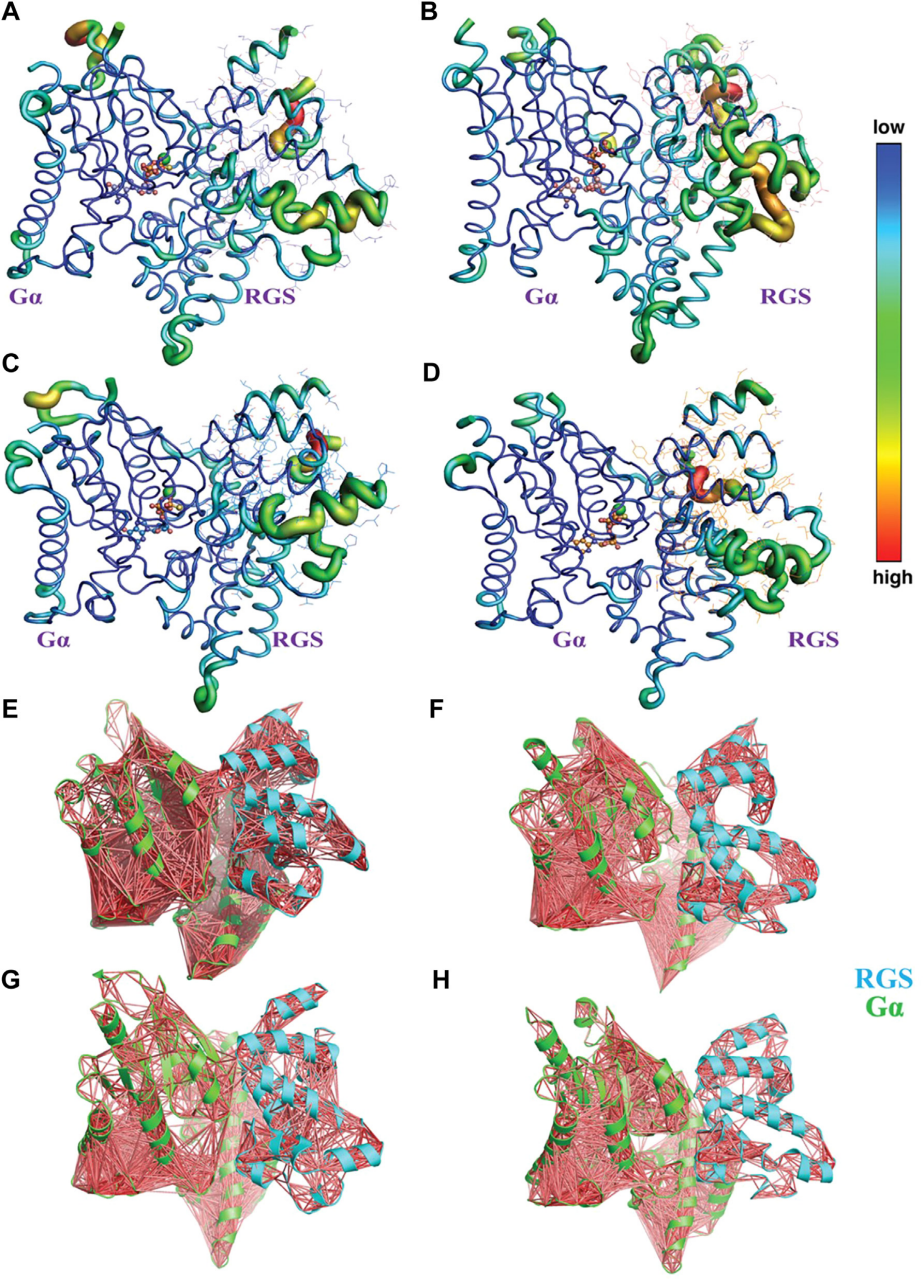
**MD simulation of plant Gα·RGS protein complexes.** Root mean square fluctuation (RMSF) is shown in color bar representation for (*A*) SiGPA·SiRGS, (*B*) AtGPA1·AtRGS, (*C*) SiGPA(N194T)·SiRGS(S321T), and (*D*) AtGPA1(T194N)·AtRGS(T321S). Gα is shown in a tube and the RGS protein in the tube and lines with color corresponding to motion, as indicated by the color bar on the *right*. Dynamic cross-correlation calculation of the degrees of motion of atoms that move together are pictorially represented for (*E*) SiGPA·SiRGS, (*F*) AtGPA1·AtRGS, (*G*) SiGPA(N194T)·SiRGS(S321T), and (*H*) AtGPA1(T194N)·AtRGS(T321S) with Gα and RGS-protein colored *green* and *cyan*, respectively.

The root mean square fluctuations (RMSF), indicating the dynamic motions of the atoms, show that RGS protein is less stable than Gα in both *A. thaliana* and *S. italica* complexes; however, the RGS protein is more flexible in *A. thaliana* than in *S. italica* (Fig. 6, *A* and *B*). These differences in the mobility of the RGS with respect to Gα could be attributed to the Asn194 of SiGPA because all other residues at the interface of the Gα:RGS complex are conserved with AtGPA1 (21). To test whether the substitution of threonine to asparagine in the SiGPA is compensated by an adaptive mutation of threonine to serine mutation in SiRGS (28), simulations using mutated AtGPA1(T194N)·AtRGS(T321S) and SiGPA(N194T)·SiRGS(S321T) complexes were performed. MD simulations of the mutated Arabidopsis complex did not show any evidence of destabilization associated with substitutions of these residues (Fig. 6, *C* and *D*). Furthermore, the dynamic cross-correlation analysis of the Gα:RGS complexes revealed that the motion between the two proteins is highly coupled in the SiGPA:SiRGS complex, but not in the AtGPA1:AtRGS complex, suggesting differences in the dynamics of the complexes between species. In the SiGPA·SiRGS complex, there is a strong correlation in the complex fluctuations, indicating that the movements of the atoms in both proteins are connected (Fig. 6, *E*–*H*). Our results suggest that the differences in the dynamics of the complexes could not be attributed to the variability of specific residues, as was proposed for Thr194 in AtGPA (11). Instead, the overall changes in the three-dimensional structure of both proteins likely influence the interaction between Gα and RGS among plant species.

## Discussion

The G-protein constituents and their regulation in plant lineages represent a major deviation from what has been established based on studies in metazoan systems (37). Although the protein complex is essential in many plants (*e.g.*, rice and maize (38, 39)), other plants tolerate the loss of one or more members with little or no consequence on fitness (29). The most glaring example is the Gα:RGS protein complex. Many algae have lost both proteins, and most bryophytes also do not have either a canonical Gα (but have a plant-specific variant) or RGS. Additionally, there are no examples where an RGS coding gene exists in the genome without a Gα coding gene, but the opposite situation is common; RGS is lost repeatedly in all plant groups (*e.g.*, bryophytes, gymnosperms, and monocots) except eudicots, which have Gα and functional G-protein signaling (2). It is intriguing that RGS, when present, is active and the Gα proteins have maintained their ability to be accelerated by RGS, regardless of the presence of a cognate RGS in the genome; but loss of RGS has no obvious effect on plant fitness. To address this, we took a structure-based approach and considered several possible hypotheses:

Hypothesis 1 **The Gα proteins of plants that have a cognate RGS protein in their genome are inherently different from those that do not, making RGS dispensable for certain species.** We show that despite varying degrees of sequence divergence, the structures of Gα proteins from a lycophyte (*S. mollendorfii*), a monocot (*O. sativa*), and a dicot (*A. thaliana*) showed almost complete conservation of the Ras-like GTPase domain (Fig. 1, Table 1). In fact, the three-dimensional structure is conserved across the plant evolutionary lineage (Fig. S2). The availability of plant Gα structures in both GDP and GTP-bound forms (Fig. 1) also showed that the conformational changes in the three key switch regions of the GTP- *versus* GDP-bound forms (Figs. 1 and S1) are similar to what is described for metazoan Gα proteins (17, 18, 19, 24). In the active GTPγS-bound structure, the conformation of the three switch regions favors interaction with the RGS protein, which is also confirmed by ITC analysis of the SmGPA:SmRGS interaction (Fig. 4). In the inactive GDP-bound structure, these same residues are disordered, which disrupts RGS interaction. This suggests that all plant Gα, in their active conformation, can interact with RGS. This is also supported by our previous work in which we have shown that the overall biochemistry of proteins from different plant groups is similar (21). Moreover, a rice Gα can fully complement all phenotypes of Arabidopsis Gα knockout (*gpa1*) mutants (30). Therefore, the hypothesis that Gα from various plants is inherently different is not supported.

Hypothesis 2 **The three-dimensional interface of Gα which interacts with RGS is different in plants without RGS protein.** Surface rendering of the SmGPA⋅GTPγS and OsRGA1⋅GDP complexes showing that the amino acids inferred to be a part of the RGS interaction region (Fig. 2) are almost fully conserved among different plant species and are similar to human Gαi. Targeted mutagenesis of these residues on SmGPA, especially Q214, N216, E217, K220, and E246 weakened their interactions with RGS and diminished RGS-dependent GTPase activity (Fig. 3). Similarly, alanine substitutions of the corresponding residues in SmRGS (E313 and N357) resulted in a loss of GAP activity and interaction with SmGPA (Fig. 5), substantiating the predictions from the structural model. As the amino acids involved in Gα:RGS are conserved between plants with or without RGS, there is no support for the hypothesis of an altered interaction interface of different plant Gα. Our previous *in vitro* biochemical data also align with these results (21).

Hypothesis 3 **A specific amino acid substation in Gα dictates the loss of RGS proteins.** MD simulation analysis of the Gα-RGS protein complexes of Arabidopsis and Setaria addressed the roles of specific amino acids proposed to be crucial for their interaction. We did not find any difference between the wild-type and mutated forms of Gα and RGS for both species (Fig. 6). While Thr194 (Thr192 in SmGPA) plays a crucial role in establishing contacts with RGS, other residues also contribute significantly to maintaining the stability of the complex, making the interaction possible regardless of the substitution of Thr194. Moreover, the overall conformation of Gα, particularly the switch regions of the Ras-like domain, is not significantly affected by the substitution of this Thr. The interactions are possibly affected by a combination of several amino acids, including those identified in this work (T192, Q214A, N216A, E217A, K220A, and E246A in SmGPA), which influences the overall conformation of Gα. We do not find any support for the hypothesis suggesting a single amino acid substitution-based loss of Gα:RGS interaction.

In conclusion, the prevailing hypotheses of why RGS is lost in several plant groups are not supported by empirical data. Our detailed evolutionary analysis has identified multiple losses of RGS in different lineages (2), and we have failed to determine any specific patterns of RGS loss. Our structure/function data are inherently consistent with additional *in vitro* and *in vivo* data. Especially, we have shown that if RGS is introduced in plants that do not naturally have it, it functions as expected and increases the GTPase activity of the native Gα protein (29).

Recent developments in plant G-protein signaling have identified several unique regulatory mechanisms. For example, the plant Gα interacts with receptor like kinases, and can be affected by de/phosphorylation, bypassing the canonical GTP/GDP-based activation (15, 17, 18). Additionally, mutations in the active site of Gα, which makes the protein completely inactive in the context of GTP-binding and hydrolysis, seem to have no or little effect on their in-planta function (40). Furthermore, the plant-specific extra-large Gα, which do not function by conventional mechanisms (41), play a major role in plant G-protein signaling to work with the canonical Gβγ proteins, that is, the loss of Gα is tolerated in plants, but many plants cannot survive without the extra-large Gα or Gβ (38, 39, 42, 43). It is possible that the plant Gα:RGS complex is present and functional in specific contexts only, or there are additional, yet unknown proteins that compensate for RGS function. A more provocative hypothesis is that alternative signaling conduits comprising receptor-like kinases, extra-large Gα proteins have evolved to function with canonical G-protein networks, and the role of not only RGS but also the canonical Gα is increasingly becoming redundant for plant physiology and function, although the proteins maintain their inherent properties when present.

## Experimental procedures

### Cloning and site-directed mutagenesis

Full-length cDNA sequences corresponding to *S. moellendorffii* Gα (SmGPA, GenBank: XP_002960996.1), *O. sativa* Gα (OsRGA1 GenBank: AAC41657.1), and the RGS domain of *S. moellendorfii* (SmRGS GenBank: XP_024539565.1; residues 250–493) were cloned into pET-28a for expression as N-terminal hexahistidine-tagged recombinant proteins in *Escherichia coli* BL21(DE3), as previously described (21). Site-directed mutants of SmGPA (D125A, Y126A, V189A, T192A, Q214A, N216A, E217A, R219A, K220A, F245A, and E246A), and SmRGS (E313A, E355A, and N357A) were generated using the QuikChange PCR method (Agilent Technologies) with the pET28a-SmGPA and pET28a-SmRGS constructs as templates, respectively.

### Protein expression and purification

Transformed *E. coli* BL21(DE3) cells containing expression constructs were grown at 37 °C in Terrific broth with 50 μg mL^−1^ kanamycin until A_600nm_∼0.6 to 0.9. After induction with 1 mM isopropyl-1-thio-β-D-galactopyranoside (IPTG), cells were grown at room temperature for 16 to 20 h. Following centrifugation (10,000*g* for 15 min), cell pellets were resuspended in 50 mM Tris, pH 8.0, 500 mM NaCl, 25 mM imidazole, 10% (v/v) glycerol, and 1% (v/v) Tween-20. Following lysis by sonication, cell debris was removed by centrifugation (35,000*g* for 1 h) and the supernatant was loaded onto a Ni^2+^- nitriloacetic acid (NTA) column (Qiagen). The column was washed with 50 mM Tris, pH 8.0, 500 mM NaCl, 25 mM imidazole, and 10% (v/v) glycerol to remove unbound proteins. Protein was eluted from the column using 50 mM Tris, pH 8.0, 10% (v/v) glycerol, 250 mM imidazole, and 500 mM NaCl. For protein crystallization, the His-tagged protein was incubated in Spectra/Por 1 dialysis membrane with thrombin (1/2000 ratio) in wash buffer overnight at 4 °C and then passed over a mixed Ni^2+^-NTA/benzamidine Sepharose column to remove uncut protein and the protease. The thrombin-cleaved protein was further purified by size-exclusion chromatography on a Superdex-200 26/60 HiLoad FPLC column equilibrated with 50 mM Tris, pH 8.0, 100 mM NaCl, 5% (v/v) glycerol, 2 mM D/L-dithiothreitol (DTT), and 1 mM MgCl_2_. Expression and purification of SmGPA mutants used a similar procedure. Preparation of the SmRGS protein for biochemical experiments was as previously described (21). All mutant proteins were expressed and purified to levels comparable to the native proteins. Protein concentrations were determined at A_280nm_ using molar extinction coefficients calculated using the ProtParam tool (http://web.expasy.org/protparam).

### Protein crystallography

Protein crystals of the SmGPA·GTPγS complex were grown by the hanging drop vapor diffusion method at 4 °C in drops of a 1:1 mixture of protein (10 mg mL^−1^) and crystallization buffer (20% (w/v) PEG-3350, 200 mM potassium citrate tribasic, 1 mM MgCl_2_, 4.3 mM GTPγS). Crystals of the OsRGA1·GDP complex were grown by the hanging drop vapor diffusion method at 4 °C in drops of a 1:1 mixture of protein (10 mg mL^−1^) and crystallization buffer (30% (w/v) PEG-4000, 100 mM Tris (pH 8.5), pH 8.0, 200 mM sodium acetate; 1 mM MgCl_2_, 5 mM GDP). For data collection, crystals were stabilized in cryoprotectant (mother liquor with 30% (v/v) glycerol) before flash freezing in liquid nitrogen for data collection at 100 K. Diffraction data were collected at beamline 19-ID of the Advanced Photon Source at the Argonne National Lab. HKL3000 was used to index, integrate, and scale the collected x-ray data (44). Molecular replacement was used to solve the x-ray crystal structures of the SmGPA·GTPγS and OsRGA1·GDP complexes using PHASER (45) with a homology model of each Gα protein as search model. Homology models of SmGPA and OsRGA1, were generated with Phyre2 (46). COOT (47) and PHENIX (48) were used for iterative rounds of model building and refinement, respectively. Data collection and refinement statistics are summarized in Table 1. The final model of the GmGPA·GTPγS complex included Asn36-Ser371 of chain A, Lys35-Ser371 of chain B, 1 GTPγS and 1 Mg^2+^ ion in each chain, and 141 waters. The final model of OsRGA1·GDP complex included His40-Asp103, Tyr107-Tyr129, Lys134-Pro204, Tyr215-Val220, Val237-Ile314, Glu320-Pro342, and Arg347-Arg375 of chain A, Ile39-Leu61, Ala70-Ser121, Tyr129-Arg155, Cys164-Pro204, Val214-Ile314, and Glu320-Arg375 of chain B, His40-Val100, Tyr107-Ile123, Pro130-Pro204, Tyr215-Val220, Val237-Ala315, and Val321-Arg375 of chain C, His40-Thr65, Glu71-Leu120, Arg126-Arg155, Pro162-Pro204, Val214-Ala315, Glu320-Ser340, and Arg347-Arg375 of chain D, and 1 GDP and 1 Mg^2+^ ion in each chain. Atomic coordinates and structure factors for the SmGPA·GTPγS (PDB: 8VGA) and OsRGA1·GDP (PDB: 8VGB complexes were deposited in the RCSB Protein Data Bank (www.rcsb.org).

### Alphafold analysis

Protein sequences of G⍺ subunits from *Arabidopsis trichopoda*, *B. distachyon*, *S. italica*, *Arabidopsis caerulea*, and *C. braunii* were obtained from Uniprot (https://www.uniprot.org), and the respective structures were predicted using AlphaFold (49, 50).

### Yeast two-hybrid assay

To determine the interaction between SmGPA and SmRGS, a GATEWAY-based yeast two-hybrid assay was performed (ProQuest Two Hybrid System, Invitrogen). SmGPA was cloned into pDEST32 bait vector and SmRGS cloned into pDEST22 prey vector. Both vectors were co-transformed into *Saccharomyces cerevisiae* host strain MaV203 (Invitrogen) according to the manufacturer’s instructions. Interactions were determined by cell growth on control and selective media (51). The experiment was repeated three times with similar results.

### GTPase activity measurement

Determination of the intrinsic and RGS-dependent GTPase activity of Gα was performed with enzyme-coupled EnzChek (Life Technologies, Carlsbad, CA) assay using 2-amino-6-mercapto-7-methylpurine riboside and purine nucleoside phosphorylase for inorganic phosphate detection, as previously described (21). Briefly, SmGPA (1 μM; wild-type or mutant) was incubated with and without SmRGS, and then phosphate (P_i_) release was measured for 30 min at A_360nm_ after the addition of 1 mM GTP. The experiments were performed three times, with three technical replicates in each experiment.

### Isothermal titration calorimetry

For ITC analysis of SmGPA-SmRGS interaction, purified proteins were dialyzed separately overnight in 50 mM Tris·HCl (pH 8.0), 100 mM NaCl, 5 mM MgCl_2_, 100 mM GDP, 30 mM AlCl_3_, 20 mM NaF, 2 mM Tris (2-carboxyl) phosphine (TCEP), and 5% (v/v) glycerol. All ITC experiments were performed at 20 °C using a VP-ITC calorimeter (Microcal, Inc). The concentration ratio of proteins in the microsyringe *versus* the cell was 10:1 with injections (10 ml) of SmGPA (wild-type or mutant) protein added to the sample solution containing SmRGS protein every 5 min by a computer-controlled 250 μl micro-syringe. Control experiments were conducted with a buffer to determine the heat of dilution for each injection. This buffer control was then applied as a correction factor in the experimental titrations. Data obtained from the titrations were analyzed using a single-site binding model, Q_i_^tot^ = V_o_·M_i_^tot^((n*K*_1_x)ΔH_1_)/(1 + *K*_1_x)), and fitted using a modified version Origin software provided by the instrument manufacturer (Microcal, Inc). Values for the change in Gibbs free energy (Δ*G*) were calculated using the equation ΔG = −RTln(*K*_eq_), where R represents the gas constant (1.9872 cal K^−1^ mol^−1^) and T is the absolute temperature in Kelvin. Changes in entropy (ΔS) were determined using the equation ΔG = ΔH-TΔS. The dissociation constant (*K*_d_) was calculated as the reciprocal of *K*_*e*q_ (*K*_d_ = 1/*K*_eq_). The experiment was performed three times, independently.

### MD simulation

MD simulations were performed with models of the *S. italica* SiGPA·SiRGS and AtGPA1-AtRGS complexes, based on the x-ray crystal structure of the human G_iα1_·RGS4 complex (22). In addition, simulation of mutated AtGPA1(T194N)·AtRGS(T321S) and SiGPA(N194T)·SiRGS(S321T) complexes were performed. For each complex, the GTPγS·Mg^2+^-bound G-protein was overlaid on the position of the human enzyme and homology models of the plant RGS superimposed on the human RGS protein, as previously described (21). GTPγS was parameterized using the RED server (52). To ensure a complete three-dimensional structure, the N- and C-termini were capped with neutral terminal residues, specifically acetyl and methyl amide groups, respectively. Hydrogen atoms were added to protein residues using the “reduce” command of the AMBER18 package (53). The tleap package of AMBER18 was used to create the simulation box with explicit solvent and all systems were solvated using the TIP3P water model (54). To neutralize the MD system, a salt concentration of 150 mM NaCl was added. MD simulations were performed using the AMBER18 with the Amber ff14SB force field parameters (55). During the MD simulation, all systems were subjected to 20,000 steps of energy minimization using a two-step minimization process with the first 5000 steps using gradient descent and the last 15,000 steps using conjugate gradient algorithm. The minimized systems were gradually heated from 0 to 300 K over a period of 2 ns in constant-temperature, constant-volume (NVT) ensembles. The Ca-atoms in the protein backbone were restrained in place by a spring force constant while temperature and pressure were being modulated. Subsequently, each system underwent 25 ns of equilibration in the NPT ensemble. The NPT ensemble maintained both the temperature and pressure at their appropriate physiological levels (*i.e.*, 300 K and 1 bar, respectively). For the production runs, we employed the NPT ensemble to simulate the systems at a constant temperature of 300 K with temperature regulated using the Langevin thermostat (56). Similarly, the pressure was kept constant at 1 atm using the Monte Carlo barostat (57). To integrate the equations of motion, a timestep of 2 fs was used. Long-range electrostatic interactions were computed using the particle mesh Ewald method (58). To address the stability issue caused by hydrogen atoms, the SHAKE algorithm was employed (59). To account for the periodic nature of the system, periodic boundary conditions were applied with a cut-off distance for the van der Waals interactions, which describes the range at which these interactions were calculated, set to 10 Å. Finally, the production runs were conducted for all the simulations for extended periods of simulation time (∼12–15 μs). Post-processing of the simulation data was performed using MDTraj (60), Pytraj (61), and Cpptraj (62).

## Data availability

All data are included in the manuscript and associated supporting information.

## Supporting information

This article contains supporting information.

## Conflict of interest

Joseph M. Jez is an associate editor and Sona Pandey is an editorial board member. The other authors declare that they have no conflicts of interest with the contents of this article.
