# Supplementary material for: Structure-function analysis of plant G-protein regulatory mechanisms identifies key Gα-RGS protein interactions
Source: J Biol Chem. 2024 Apr 1;300(5):107252. doi: 10.1016/j.jbc.2024.107252 (PMC11061236; doi:10.1016/j.jbc.2024.107252)
Supplement: Supporting Information [file mmc1.docx]

**Supporting Information**

**Structure-Function Analysis of Plant G-protein Regulatory Mechanisms Identifies Key Gα-RGS Protein Interactions**

**Maria Daniela Torres-Rodriguez^1,‡^, Soon Goo Lee^2,‡^, Swarup Roy Choudhury^1,3,‡^, Rabindranath Paul^4^, Balaji Selvam^4^, Diwakar Shukla^4^, Joseph M. Jez^5^, Sona Pandey^1,*^**

Includes Supporting Figures S1 and S2.


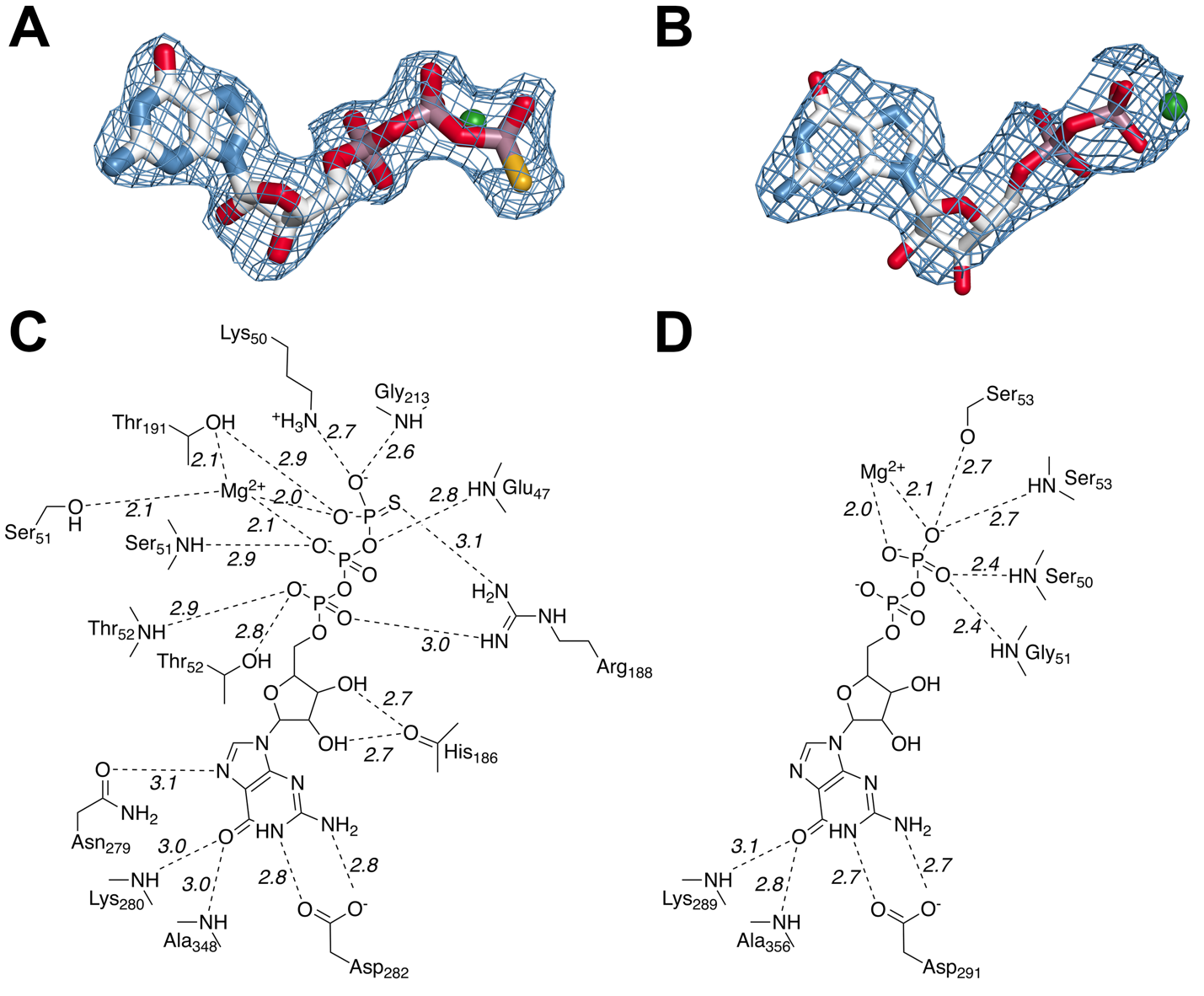


**Supplementary Figure S1.** (A) Electron density for GTPγS bound to SmGPA shown as a 2F_o_-F_c_ omit map (1.5 σ). (B) Electron density for GDP bound to OsRGA1 shown as a 2F_o_-F_c_ omit map (1.5 σ). (C) Schematic of interactions in the GTPγS binding site of SmGPA. Interactions are shown as dotted lines with distances indicated in Å. (D) Schematic of interactions in the GDP binding site of OsRGA1. Interactions are shown as dotted lines with distances indicated in Å.

**
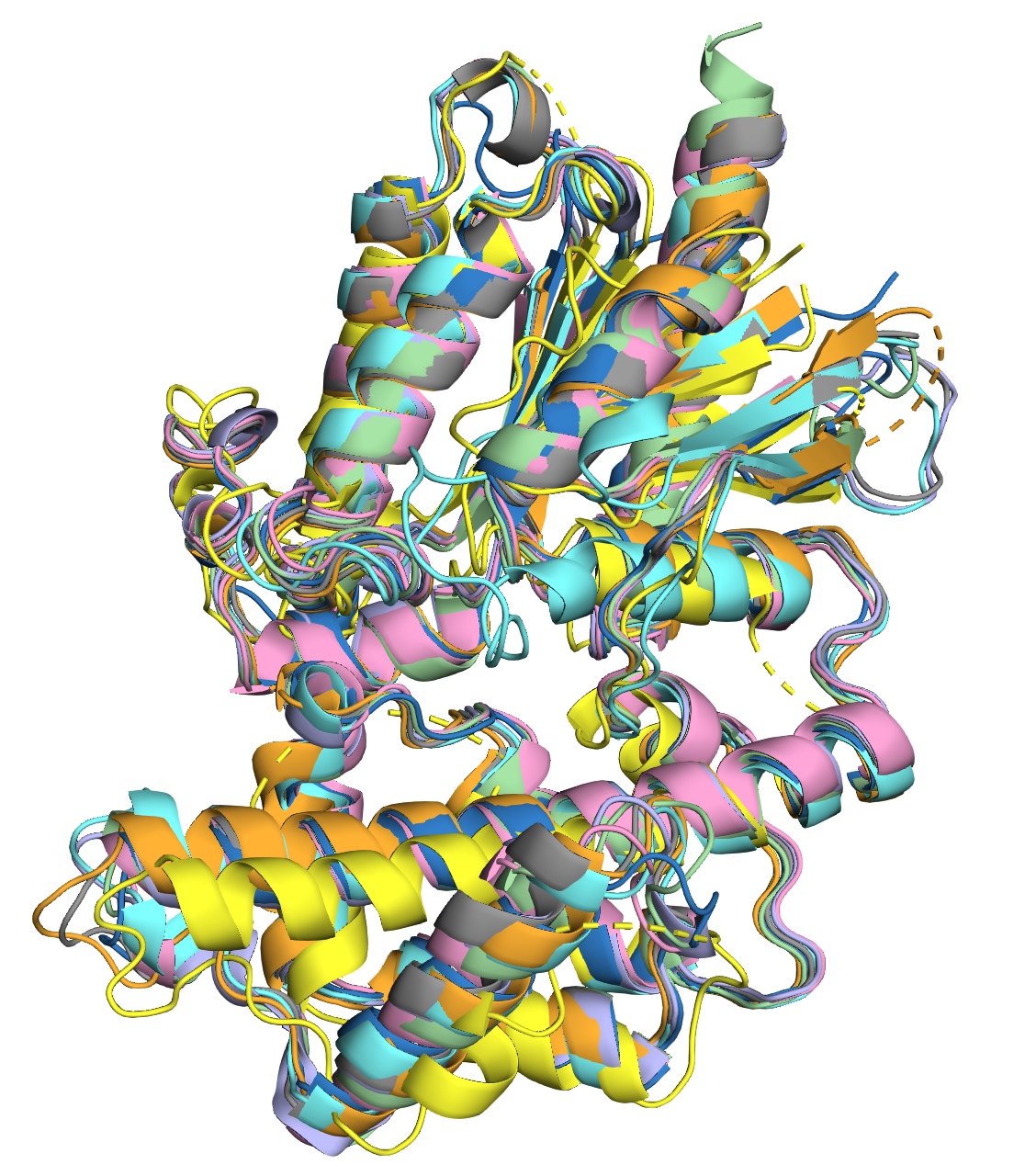
**

**Supplementary Figure S2.** Superimposition of the G⍺ structures from different plant lineages represented by *Chara braunii* (pale green) representing algae, *Marchantia polymorpha* (light pink) representing liverworts, *S. moellendorffii* (blue) representing lycophytes, *Amborella trichopoda* (gray) representing basal angiosperms, *Aquilegia caerulea* (light purple) and *A. thaliana* (light orange) representing eudicots, and *O. sativa* (yellow) and *S. italica* (cyan) representing monocots, without or with a cognate RGS, respectively. The structures were predicted using AlphaFold2.
